# The Standard Dictionary of Funk & Wagnalls

**Published:** 1896-07

**Authors:** 


THE STANDARD DICTIONARY of Funk & Wagnalls.

On the table side by side at the Wadsworth House, Cambridge, Mass., the home of the clergy of Harvard, lie peaceably two of the great rival dictionaries, the Webster International and the Funk & Wagnalls Standard. In the former is this inscription:

"To the Harvard University, for the use of the staff of University preachers—'for the correction of their English.'

"PHILIP S. MOXON,
"Feb. 28th, 1895. of the Staff of 1894–5."

This caught the eye of Bishop Vincent, who presented a copy of the Standard with the following inscription:

"To the Harvard University, for the use of the staff of University preachers, thinking that *the very best* is not too good for them.

"JOHN H. VINCENT,
"April 8th, 1895. of the Staff of 1893–5."

